# Management and endovascular therapy of ureteroarterial fistulas: experience from a single center and review of the literature

**DOI:** 10.1186/s42155-021-00226-6

**Published:** 2021-04-17

**Authors:** Bjoern Simon, Jakob Neubauer, Martin Schoenthaler, Simon Hein, Fabian Bamberg, Lars Maruschke

**Affiliations:** 1 Department of Radiology, Medical Center – University of Freiburg, Faculty of Medicine, University of Freiburg, Freiburg, Germany; 2 Department of Urology, Medical Center – University of Freiburg, Faculty of Medicine, University of Freiburg, Freiburg, Germany; 3 Diagnostic Radiology, Pediatric Radiology and Interventional Radiology, St.-Josefs-Krankenhaus, Freiburg, Germany

**Keywords:** Ureteroarterial fistula, Arterioureteral fistula, Endovascular therapy, Hematuria, Ureteral catheterization, Stent graft, Coil embolization

## Abstract

**Background:**

Ureteroarterial fistula (UAF) is a rare but potentially life threatening disease. The aim of this study was to evaluate the outcome of endovascular therapy for UAF treatment.

**Methods:**

This retrospective case series evaluates a single center experience of percutaneous stent graft (SG) angioplasty and/or coil embolization for UAF. Patient follow-up included technical and early clinical success, complications and revisional procedures. We also conducted a systematic review of the literature reporting on endovascular UAF management.

**Results:**

We identified 17 UAF in 16 patients (12 male, 4 female, mean age 69.8 ± 11.3 years) who underwent endovascular UAF therapy at our tertiary hospital. All patients presented with hematuria. 5/17 (29.4%) presented with flank pain, in 7 (41.2%) cases patients were in hypovolemic shock. Risk factors of UAF included chronic indwelling ureteral stents in all fistulas, major pelvic surgery in 13 cases (76.5%). In 6 cases (35.3%) SG were placed from the common iliac artery (CIA) to the external iliac artery (EIA) following coil embolization of the proximal internal iliac artery (IIA). SG placement without previous coil embolization was performed in 10 fistulas (58.8%). In one case only coil embolization of the IIA was performed. Mean follow-up was 654 (range: 1–3269) days. All procedures were technically successful and no procedure related deaths occurred during follow-up. During the initial hospital stay hematuria disappeared in 14/17 cases (82.4%). Overall, four patients suffered recurrent hematuria, which in three cases resolved after a secondary intervention. One recurrent UAF related death occurred during follow-up 229 days after initial treatment.

A total of 152 UAF cases were additionally analyzed from our systematic literature review: SG placement with or without embolization was performed in 140 cases (92.1%) while embolization alone was done in 12 cases (7.9%). Complications included UAF recurrence (18/152, 11.8%), SG thrombosis (7/140, 5%), and SG infections (5/140, 3.6%) with an overall complications rate of 13.8%. Five patients died due to UAF (3.3%).

**Conclusion:**

Endovascular therapy offers high technical success rates and rapid bleeding control of UAF. Severe complications like SG occlusions or SG infections are rare but significant. Antibiotic treatment and single anti-platelet therapy improve SG durability as well as close and long follow-up to timely perform repeated endovascular or surgical treatment if necessary.

**Evidence-based medicine:**

Level 4, case series.

## Background

Ureteroarterial fistula (UAF) is a rare but potentially life threatening clinical entity first described in 1908 by Moschcowitz (Moschcowitz, 1908). UAF is classified into primary (15%) and secondary (85%) lesions (Pillai et al., 2015). Primary fistulas are mainly seen in combination with aortoiliac aneurysmal disease. Secondary fistulas typically occur after pelvic surgery for malignancy with or without radiation therapy, or after vascular surgery with synthetic grafting (Bergqvist et al., 2001; Luther et al., 2014). The most common risk factor to develop an UAF is the presence of a chronic indwelling ureteral stent (Das et al., 2016). This is possibly due to the fact that mechanical fixation of the ureter triggers inflammation and fibrosis in the adjacent pulsating artery (Horie et al., 2019).

The most common location of UAF is the crossing of the distal left common iliac artery (CIA) and the ureter (Das et al., 2016; van den Bergh et al., 2009). The leading symptom is gross hematuria (Bergqvist et al., 2001; Das et al., 2016; Krambeck et al., 2005). UAF is a rare condition; however, since the increase in life expectancy in patients suffering of pelvic malignancies, this condition is observed more often (Das et al., 2016; Fox et al., 2011).

Different treatment options including surgery or surgery combined with transarterial embolization have been described (Fox et al., 2011; Krambeck et al., 2005; van den Bergh et al., 2009). More recently, stent graft (SG) placement has been reported as an effective alternative treatment; however, only small series using a variety of different, mostly self-expanding SG or iliac extensions from aortic bifurcation grafts have been reported (Fox et al., 2011; Guntau et al., 2017; Krambeck et al., 2005; Okada et al., 2013).

Thus, the purpose of the study was to evaluate the safety and effectiveness of endovascular therapy in UAF and to establish an interventional radiological therapy regime for these rare but critical cases.

## Methods

IRB approval was obtained on June 25, 2020 by the institutional review board of the University of Freiburg (#368/20).

We included all consecutive patients referred for endovascular therapy for UAF at our institution from November 2005 to March 2020. We reviewed patient characteristics, comorbidities, and clinical presentation from medical records. The clinical diagnosis of an UAF was based on pulsatile ureteral bleeding from either side on cystoscopy and/or in retrograde pyelogram by the referring urologist.

Radiological diagnostic work up included either contrast-enhanced computed tomography (CECT), unenhanced computed tomography (UECT) or conventional angiography. CT was used to rule out renal hemorrhages and plan endovascular treatment. All patients underwent conventional catheter angiography. Angiographic procedures were performed under local anesthesia via a percutaneous transfemoral approach. Five French (Fr) angiographic sheaths were placed using Seldinger’s technique. Angiograms were obtained with manual contrast injection via a 5 Fr angiographic catheter. For SG placement, the 5 Fr sheath was replaced by a larger sheath (7 Fr – 14 Fr) and an additional stiff wire was used. Most UAF were treated with percutaneous covered SG placement into the iliac axis covering the site of the fistula. In a minority of cases fistulas we performed an additional coil embolization of the internal iliac artery (IIA). Stent size was chosen to match the individual patient’s anatomy. In one patient the bleeding site was localized in the IIA alone. In this case we treated the fistula by coil embolization of the IAA without SG placement. The procedure was considered complete and technically successful after a final angiogram confirmed the correct SG placement at the target location and/or the sufficient embolization of the IIA. Early clinical success was defined as freedom of hematuria in the current hospital stay. Patients were routinely followed at our outpatients clinic or by private urologists as all patients carry ureteral stents. Follow-up examinations are performed according to individual requirements (minor bleeding episodes, plugging of ureteral stents, hydronephrosis; usually 2 to 4 weeks after ureteral stent placement, followed by routine exams every 3 to 6 months). Follow-up examinations include urinalysis, urine culture, laboratory tests, and sonography. Periinterventional management included antibiotic treatment based on urine cultures and resistance profiles. Since all patients carried ureteral stents, we found no patient without bacterial colonization. Anticoagulation therapy was switched to low-molecular weight heparin equivalent to the patients‘baseline anticoagulation dosage or prophylactic dosage. The decision for postinterventional anti-platelet therapy was implemented in consultation with the referring urologists and intensive care physicians. All but two patients were put on acetylic salicylic acid 100 mg/day immediately following UAF treatment, 6 patients received prolonged dual antiplatelet regime of additional clopidogrel 75 mg/day (12 weeks) and short-term dual antiplatelet therapy (clopidogrel 75 mg/day for 6 weeks) in one case. One patient received 12 weeks of clopidogrel 75 mg/day combined with previously started oral anticoagulation. The patient with coil embolization only did not receive any anti platelet therapy. A descriptive analysis was performed. Continuous data were summarised by median, arithmetic mean, and range. Categorical data were summarised by the total number of patients in each category. Relative frequencies are displayed as valid percentages.

We performed a literature search of the PubMed database using the following keywords: “ureteroarterial fistula”, “arterioureteral fistula” and “uretero iliac fistula”. We identified 55 articles published in English from January 2018 to March 2021. Studies were included if they presented data of adult patients regarding endovascular therapy, results on technical success, complications, and outcomes of UAF. Articles were excluded if they reported nonextractable data, UAF treatments other than endovascular, or were missing data on outcome or management of UAF. We also excluded systematic reviews, comment articles, or correspondence.

## Results

In the study period, 17 endovascular UAF cases with a total of 16 patients from the author’s hospital were analyzed.

Furthermore, we updated the comprehensive review by Subiela et al. (Subiela et al., 2018), who published a case series of their own patients and from a systematic literature review on articles regarding the endovascular management of UAF between 1990 and 2017. From the 55 identified articles through PubMed database research, we included only studies which were considered of interest and met the inclusion criteria. Finally, 15 articles were added to the review by Subiela et al. with a total of 152 UAF cases (Table 1).

**Table 1:** Review of the literature

Reference	Cases	UAF treatment	Revisional procedure	Results	Complications	UAF recurrence	Follow-up and outcomes
(Subiela et al., 2018)	94	89 (94.7%) SG, 5 (5.3%) embolization, 24 (22.6%) IIA embolization + SG	nephrectomy (3 cases), SG placement (3)	In all cases, after the procedure, hematuria disappeared	overall complication rate (17%), SG thrombosis (3 [3.2%] cases), retroperitoneal abscess (2 [2.1%] cases), urosepsis (2 [2.1%] cases), native iliac external artery thrombosis (1 [1%] case), and limb claudication (1 [1%] case)	7 cases (7.5%)	overall survival: 42 months (95% CI: 32.4–51.6), survival (UAF related death): 2 months (95% CI: 0–15)
(Devulapalli et al., 2021)	1	IIA vascular plug and coil embolization	no	technical success	no	no	free of gross hematuria at 2.5 years
(Kaneko et al., 2020)	1	IIA coil embolization + SG	no	technical success	no	no	died seven months after treatment unrelated to UAF
(Augustin et al., 2020)	5	2/5 (40%) coil embolization, 3/5 (60%) vascular plug embolization, 2/5 (40%) SG	coil and microspheres (Embozene) embolization and additional open-surgical occlusion (1 case); SG extension (1)	100% technical success, 60% clinical success	fever after a urological exchange of an ureter stent (1 case)	2/5 (40%), 8 days and 4 days after initial therapy	Median follow-up: 39 (range: 1–48.7) months
(Yoshioka et al., 2020)	1	IIA vascular plug embolization + SG	no	technical success	no	no	uneventful at 11 months
(Fernandopulle et al., 2020)	1	SG	no	technical success	no	no	uneventful at 9 months
(Geevarghese and Gupta, 2020)	1	SG	no	technical success	no	no	uneventful at 6 months
(Perrenoud et al., 2020)	1	SG	two times SG extension	technical success	febrile, suspected SG infection	within a week and again 4 days after repeated intervention	continued to have intermittent hematuria and ultimately required surgical revision
(Berastegi-Santamaria et al., 2020)	1	SG	no	technical success	no	no	uneventful at 1 month
(Di Grazia et al., 2020)	1	SG	no	technical success	no	no	uneventful at 1 month
(Massmann et al., 2020)	5	SG in a double-barrel technique,	1/5 (20%) femoral-femoral-crossover bypass	100% technical success	1/5 (20%) SG thrombosis at 6 weeks	no	median follow-up: 18 (9–37) months
(Horie et al., 2019)	1	IIA coil embolization + SG	no	technical success	no	no	uneventful 1 year
(Titomihelakis et al., 2019)	5	3/5 (60%) SG, 2/5 (40%) IIA coil embolization + SG	1/5 (20%) SG, 1/5 (20%) surgery	4/5 (80%) successful repairs	2/5 (40%) SG infections, 1/5 (20%) SG occlusion	2/5 (40%)	1 lost to follow-up, 1 died at one year unrelated to UAF treatment, 1 uneventful at 10 months, 1 without hematuria at 7-month follow-up, 1 died 5 months due to rebleeding
(Leone et al., 2019)	4	SG	no	technical success	no	no	Mean follow-up: 49 (25–66) months, uneventful in all patients
(Heers et al., 2018)	22^a^	17/22 (77.3%) SG, 3/22 (13.6%) coil embolization + SG, 2/22 (9.1%) coil embolization,	SG (2 cases), coil embolization (later surgical ligation and cross-over bypass; 1 case)	24/26 (92.3%) survived the acute situation	2 SG occlusions, 1 SG infection, 1 ischemic pain in ipsilateral hip, 1 ipsilateral calf ischemia	3/26	2/26 (7.7%) did not survive the acute situation, one was lost to follow-up, 12 were alive at a median follow-up of 8 months (1–80), nine died during follow-up (one died due to ongoing UAF)
(Noh et al., 2020)	8	6/8 (75%) SG, 1/8 (12.5%) SG + IIA embolization, 1/8 (12.5%)^b^	3/8 (37.5%; surgery [2], SG [1])	100% technical success, no UAF-related death	1/8 (12.5%) SG infection	3/8 (37.5%) at a mean of 6.3 months	median follow-up: 987 days, two died due to their underlying disease (45 and 288 days)

*IIA* internal iliac arery, *SG* stent graft, *UAF* ureteroarterial fistula

^a^ 22 endovascular cases of 26 UAF including 4 surgical cases, ^b^ ureteral occlusion stent, embolization with coils and cyanoacrylates

### Clinical findings

In the study period, 17 UAFs with a total of 16 predominantly male patients were included in the analysis (75% male, mean age 69.8 ± 11.3 years). All patients presented with hematuria. Approximately one third of patients (31.3%) exhibited additional flank pain, in seven cases (41.2%) patients were in hypovolemic shock. In 94.1% of the cases patients presented with acute urinary retention. Half of the patients had chronic nephropathy. Risk factors of UAF included chronic indwelling ureteral stents in all fistulas, major pelvic surgery in 13 cases (76.5%) and history of pelvic or genitourinary malignancies including colorectal cancer (10 cases), uterine/cervical cancer (3 cases), bladder cancer (1 case), testicular cancer (1 case) and prostate cancer (1 case). One patient had radiotherapy as primary treatment, one patient was under active surveillance and the only patient who did not suffer of a malignancy but of Ormond’s disease had cortisone therapy as primary treatment. Fourteen of 15 patients with cancer had adjuvant radiation therapy and chemotherapy. Five patients (31.3%) suffered of vasculopathies, two had severe claudication (Rutherford III), one had a right critical limb ischemia (Rutherford V), one had abdominal aortic aneurysmal disease and one patient had documented peripheral arterial occlusive disease with aortofemoral bypass. Patient characteristics are summarized in Table 2.

**Table 2:** Clinical findings

Case	Patient	Age (years)	Sex	Underlying disease	Treatment of primary disease	UAF risk factors	Symptoms	Vasculopathy
1	1	38	female	Rectal cancer	Rectum resection	Radiation therapy, chemotherapy, permanent ureteral catheterization	flank pain, hematuria, hypovolemic shock, urinary retention	–
2	2	81	male	Rectal cancer	Rectum resection	Radiation therapy, chemotherapy, permanent ureteral catheterization	flank pain, hematuria	–
3	3	81	female	Cervix cancer	Wertheim’s procedure	Radiation therapy, chemotherapy, permanent ureteral catheterization	flank pain, hematuria, hypovolemic shock, urinary retention	Rutherford III severe claudication
4	4	67	male	Rectal cancer	Rectum resection	Radiation therapy, chemotherapy, permanent ureteral catheterization	flank pain, hematuria, urinary retention	–
5	5	80	male	Ormond’s disease	Cortisone	permanent ureteral catheterization	flank pain, hematuria, hypovolemic shock, urinary retention	–
6	6	60	male	Rectal cancer	Rectum resection	Radiation therapy, chemotherapy, permanent ureteral catheterization	flank pain, hematuria, hypovolemic shock, urinary retention	–
7	7	83	male	Rectal cancer	Rectum resection	Radiation therapy, chemotherapy, permanent ureteral catheterization	flank pain, hematuria, hypovolemic shock, urinary retention	–
8	8	71	male	Prostate cancer	Active surveillance	permanent ureteral catheterization	flank pain, hematuria, urinary retention	Rutherford III severe claudication
9	9	61	male	Rectal cancer	Rectum resection	Radiation therapy, chemotherapy, permanent ureteral catheterization	flank pain, hematuria, urinary retention	–
10	10	58	female	Cervix cancer	Radiochemotherapy	Radiation therapy, chemotherapy, permanent ureteral catheterization	flank pain, hematuria, urinary retention	Rutherford V limb ischemia
11	11	86	female	Uterus carcinoma	Hysterectomy	Radiation therapy, chemotherapy, permanent ureteral catheterization	flank pain, hematuria, urinary retention	–
12	12	76	male	Urothel carcinoma	Cystectomy	Radiation therapy, chemotherapy, permanent ureteral catheterization	flank pain, hematuria, urinary retention	–
13	13	66	male	Sigmoid carcinoma	Sigmoid resection	Radiation therapy, chemotherapy, permanent ureteral catheterization	flank pain, hematuria, hypovolemic shock, urinary retention	–
14	14	69	male	Rectal cancer	Rectum resection	Radiation therapy, chemotherapy, permanent ureteral catheterization	flank pain, hematuria, urinary retention	–
15	15	71	male	Rectal cancer	Rectum resection	Radiation therapy, chemotherapy, permanent ureteral catheterization	flank pain, hematuria, urinary retention	abdominal aortic aneurysm
16	16	67	male	Testicular cancer	Orchectomy	Radiation therapy, chemotherapy, permanent ureteral catheterization	flank pain, hematuria, hypovolemic shock, urinary retention	peripheral occlusive disease
17	15	71	male	Rectal cancer	Rectum resection	Radiation therapy, chemotherapy, permanent ureteral catheterization	flank pain, hematuria, urinary retention	abdominal aorticaneurysm

*UAF* ureteroarterial fistula

### Imaging

Urologists were able to confirm signs of ureteral bleeding and correctly located the site of bleeding by using cystoscopy in all patients. In 10 cases the first radiological diagnosis was done by CECT, in 4 cases direct catheter angiography in readiness to intervene followed urological diagnosis confirmation, and in 3 cases an unenhanced CT was performed prior to angiography. CECT directly identified the fistula only in two cases (of 10 cases who received preinterventional CECT) with extravasation into the ureter. Angiography was positive in 3 of 17 cases. CT or angiography identified the crossing point between ureter and iliac artery in all patients. In 10 cases, the UAF was located at the CIA, in 3 cases each the IIA or the external iliac artery (EIA) was compromised respectively; in one exceptional case the fistula was located at the aortofemoral bypass graft.

### Procedures

We inserted SG without previous coil embolization in 10 fistulas. In 6 cases we performed coil embolization of the proximal IIA prior to SG implantation. In one case the UAF was located solely in the IIA and was therefore treated by coil embolization alone. In three cases a secondary procedure was necessary due to recurrent bleeding during the hospital stay: In two cases SG extension and additional coil embolization of the IIA was needed and in one fistula IAA coiling alone was sufficient. Unrelated to the UAF but because of ongoing aneurysm growth due to aneurysmosis of the aortoiliac territory a secondary SG extension into the proximal CIA was required in one case.

Different types of balloon-expandable and self-expandable SG were used: Advanta V12 (Atrium Medical, NH, USA) in 7 cases, Fluency (Bard Peripheral Vascular, Tempe, AZ, USA) in 5, Gore Excluder endograft (Gore, Flagstaff, AZ, USA) in 3, Viabahn endograft (W. L. Gore, Flagstaff, AZ, USA) in 3 and Endurant II endograft (Medtronic Vascular, Santa Rosa, CA, USA) in 1. In 16 of 17 cases puncture site was closed by a closure device: In 4 cases FemoSeal™ (St. Jude Medical, Plymouth, MN, USA) was used, in 9 cases Perclose ProGlide (Abbott Vascular, Abbott Park, IL, USA) was applied, StarClose (Abbott Vascular, Abbott Park, IL, USA) was used twice and in one case Prostar XL (Abbott Vascular, Santa Clara, CA, USA) was employed. Diagnostic and technical data are shown in Table 3.

**Table 3:** Technical data and procedures

Case	Patient	Primary radiological diagnostics	Fistula location	UAF treatment	Stent data	Revisional procedure	Medication postinterventionally
1	1	CECT	EIA	SG + IIA Coil embolization	8/38 mm Advanta V12	–	ASS100mg
2	2	CECT	EIA	SG + IIA Coil embolization	10/59 mm Advanta V12	–	ASS100mg + 12 weeks Clopidogrel 75 mg
3	3	CECT	CIA	SG	8/59 mm Advanta V12	Interventional thrombectomy	Oral anticoagulation + 12 weeks Clopidogrel 75 mg
4	4	CECT	IIA	SG + IIA Coil embolization	16/13/80 Endurant II	–	ASS100mg + 12 weeks Clopidogrel 75 mg
5	5	CECT	EIA	SG + IIA Coil embolization	13.5/80 mm Fluency	–	ASS100mg + 12 weeks Clopidogrel 75 mg
6	6	CECT	CIA	SG + IIA Coil embolization	13.5/80 mm Fluency	–	ASS100mg + 12 weeks Clopidogrel 75 mg
7	7	CECT	Obturator artery/IIA	SG	6/50 mm Viabahn; 10/59 mm Advanta V12	SG + IIA Coil embolization due to recurrent bleeding	ASS100mg + 12 weeks Clopidogrel 75 mg
8	8	UECT	CIA	SG + IIA Coil embolization	10/59 mm Advanta V12	–	ASS100mg
9	9	UECT	CIA	SG	16/12/70 mm Gore excluder;	–	ASS100mg
10	10	CECT	CIA	SG	7/50 mm Viabahn	–	ASS100mg
11	11	UECT	CIA	SG	10/59 mm Advanta V12; 13.5/80 mm Fluency	SG + IIA Coil embolization due to recurrent bleeding	ASS100mg
12	12	UECT	CIA	SG	11/100 mm Viabahn	Surgical thrombectomy	ASS100mg
13	13	CECT	IIA	IIA Coil embolization	–	–	–
14	14	CECT	CIA	SG	6/38 mm Advanta V12	Interventional thrombectomy and thrombolysis	ASS100mg + 12 weeks Clopidogrel 75 mg
15	15	Angiography	CIA	SG	16/12/70 mm Gore Excluder; 16/14/120 mm Gore Excluder	SG extension due to aneurysm growth	ASS100mg
16	16	Angiography	Aortofemoral Bypass	SG	13.5/80 mm Fluency	IIA Coil embolization due to recurrent bleeding	ASS100mg + 6 weeks Clopidogrel 75 mg
17	15	Angiography	CIA	SG	13.5/80 mm Fluency	–	ASS100mg

*CECT* contrast-enhanced computed tomography, *CIA* common iliac artery, *IIA* internal iliac artery, *EIA* external iliac artery, *SG* stent graft, *UAF* ureteroarterial fistula, *UECT* unenhanced computed tomography

In our literature review SG placement with or without embolization was performed in 140 cases (92.1%) while embolization alone was done in 12 cases (7.9%).

### Outcomes

Mean follow-up was 654 (range: 1–3269) days and four patients (23.5%) were lost to follow-up between day one and 11 days. Among the remaining cases, no primary procedure-related deaths occurred during follow-up. Similarly, 30-day mortality rate was zero. All procedures were technically successful. During current hospital stay hematuria disappeared in 14/17 cases (82.4%). After a more extensive re-do intervention hematuria resolved in the remaining cases.

Recurrent hematuria happened in four cases, three of which occurred during the current hospital stay (8, 8 and 13 days after initial treatment). Two could successfully be treated via secondary SG extension over the iliac crossing plus additional coil embolization of the IIA (case #7 and #11). In case #16 where the fistula was located at the ureteral crossing of the aortofemoral bypass graft secondary coil embolization of the IIA alone was sufficient. There was one recurrent UAF related death 229 days after initial treatment (case #3). In this patient, a re-opening of the fistula was found, caused by attempted interventional thrombectomy for critical limb ischemia. Periprocedural complications happened in two cases – in case #3 the SG could not fully be unfolded and in case #12 the Prostar XL system did not work after placement of a 14Fr-sheath which led to prolonged manual compression. SG thrombosis happened in 3 cases (1, 2 and 58 days after initial treatment). Two occlusions could have been managed via interventional thrombectomy with or without thrombolysis (case #3 and case #14). In case #12 where the failed closure device led to immediate thrombosis the day after the patient was transferred to surgical thrombectomy. Septicemia was found in 5 of 17 cases (29.4%) at the point of intervention, in follow up there was no history of SG infections detected in those patients. During follow-up, one patient died of the underlying disease (576 days after the initial treatment). Early and long-term outcomes are summarized in Table 4.

**Table 4:** Outcomes

Case	Patient	Technical success	Early clinical success	Periprocedural complication	Stent thrombosis (days)	Recurrent bleeding (days)	Follow-up data
1	1	yes	yes	–	no	no	uneventful at 4 days
2	2	yes	yes	–	no	no	uneventful at 1 days
3	3	yes	yes	Not fully unfolded stent	yes (2)	yes (229)	death at 229 days due to recurrent hemorrhage
4	4	yes	yes	–	no	no	uneventful at 3 days
5	5	yes	yes	–	no	no	uneventful at 69 days
6	6	yes	yes	–	no	no	uneventful at 186 days
7	7	yes	no	–	no	yes (8)	alive at 11 days
8	8	yes	yes	–	no	no	uneventful at 1390 days
9	9	yes	yes	–	no	no	uneventful at 846 days
10	10	yes	yes	–	no	no	uneventful at 1237 days
11	11	yes	no	–	no	yes (13)	alive at 763 days
12	12	yes	yes	Closure device failure	yes (1)	no	alive at 220 days
13	13	yes	yes	–	no	no	uneventful at 1984 days
14	14	yes	yes	–	yes (58)	no	alive at 3269 days
15	15	yes	yes	–	no	no	alive at 187 days
16	16	yes	no	–	no	yes (8)	death at 576 days due to underlying disease
17	15	yes	yes	–	no	no	alive at 147 days
Total (%)		17 (100%)	14 (82.4%)	2 (11.8%)	3 (17.7%)	4 (23.5%)	Mean follow-up 654 (Range: 1–1984) days

Follow-up data varied substantially in our systematic literature review (Table 1). UAF recurrence (18/152, 11.8%), SG thrombosis (7/140, 5%) and SG infections (5/140, 3.6%) as main complications were observed (overall complications rate 13.8%). The management of recurrent fistula was in 10 cases SG placement and in 10 cases open surgery. Two patients had embolization for UAF recurrence but finally needed surgical management, as well as one patient treated by SG extension. Five patients died related to UAF (3.3%).

## Discussion

To our knowledge we conduct the largest retrospective interventional radiological cohort study of cases with secondary UAFs from a single center.

The main symptom in our UAF patients was hematuria in all cases ranging from non-life-threatening transient hemorrhage to hypovolemic shock (in 41.2% of the cases). The need for blood transfusions and/or inotropic therapy is common. Flank pain and fever are further occurring as well (Heers et al., 2018).

**Fig. 1:**
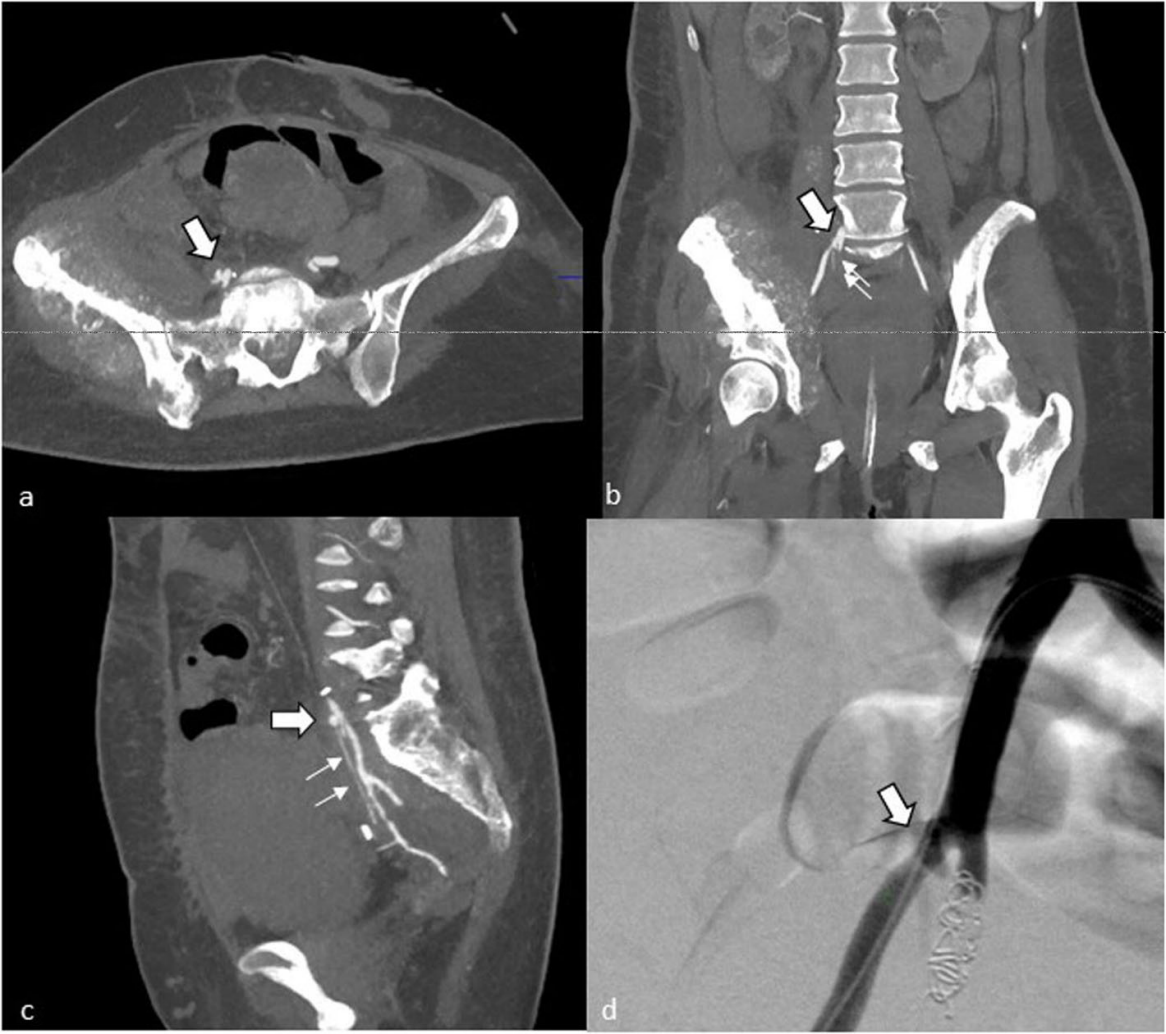
Case #1: Contrast enhanced computed tomography confirmed the ureteroarterial fistula (large arrows) and demonstrated even extravasation into the right ureter (small arrows) (**a**, **b**, **c**). Angiography shows a defect at the iliac junction (large arrow). Coil embolization of the internal iliac artery has already been performed (**d**)

Our diagnostic work up showed that in the minority of cases an active bleeding site could be located via CECT (2/10 cases; 20.0%) or angiography (3/17 cases; 17.7%). This confirms the findings of Guntau et al. with two detected UAFs in eight patients by CECT alone (Guntau et al., 2017). However, a negative CECT or angiogram does not rule out the diagnosis, therefore cystoscopy is used for confirmation of UAF and detecting the site of the fistula (Krambeck et al., 2005). Nevertheless, CECT proves to be crucial for planning the interventional approach, to locate the ureteral crossing and to rule out any other bleeding sites. Provocative angiography as an invasive imaging tool has been described as an effective procedure to demonstrate the active hemorrhage (Das et al., 2016; Fox et al., 2011; Guntau et al., 2017; van den Bergh et al., 2009). However, the risk of triggering extensive bleeding or to re-open a currently clotted fistula seems unnecessarily high. Positive CECT and angiography is shown in Fig. 1.

In the therapeutic management, there has been a paradigm shift from a surgical approach, which was still the standard of care in 2004, toward interventional angiography (Bergqvist et al., 2001; Fox et al., 2011; Madoff et al., 2004). Patients typically carry a high risk for complications due to previous extensive surgery and radiation therapy which lead to adhesions, fibrosis, and frail tissue (Heers et al., 2018). In patients with UAF to the IIA only, arterial coil embolization can be considered. Most of the UAF involve EIA or CIA, so covered stent treatment is necessary (Muraoka et al., 2006; van den Bergh et al., 2009). SG treatment is less invasive than open surgery and offers rapid bleeding control (Patel et al., 2014).

Endovascular therapy offers high technical and early clinical success rates (100% and 82.4% in our cohort). Our literature review revealed nearly 100% technical success rates through all studies (Table 1). The large patient series of 94 UAF by Subiela et al. shows disappearance of hematuria in all cases after the procedure (Subiela et al., 2018). Severe complications like SG occlusions or SG infections are rare (Malgor et al., 2012; Okada et al., 2013). Stent thrombosis is one important postinterventional complication: In our cohort we observed SG thrombosis in 17.7% of the cases in opposition to 5% in our literature review. In our experience major risk factors for developing stent thrombosis were underlying vasculopathy or advanced tumor disease with extrinsic stenosis of iliac vessels since all three patients who developed SG thrombosis had a history of pelvic malignancy with previous surgery and radiotherapy, one of them also had advanced peripheral artery disease. Massmann et al. found stent thrombosis in their case series (1/5 cases, 20%) probably caused by pre-existing large tumor vessel invasion and small vessel diameters (Massmann et al., 2020). Although we would primarily suggest an endovascular thrombectomy attempt for SG thrombosis, our series and the experience of Massmann et al. show interventional and surgical thrombectomy both to be suitable options (Massmann et al., 2020). Too maintain the risk for SG occlusions as low as possible patients should be put on single anti platelet therapy after the procedure. Periprocedural complications can be decreased by using suitable equipment, like closure devices after the use of larger sheaths. One SG thrombosis in our cohort happened due to device failure which led to prolonged manual compression and stent occlusion the day after. Literature already disclosed controversies after use of Prostar XL (Maniotis et al., 2017; Power et al., 2019).

In spite of a high number of reported urinary tract infections which sometimes lead to septicemia in these patients, the risk of secondary SG infections seems to be overrated (Darcy, 2009). In the current study none of our patients suffered from morbidity due to SG infections during their postinterventional course. In our literature research we found 5 infections of 140 implanted SG (3.6%). This matches previously published experience (Malgor et al., 2012). Since all our patients carried ureteral stents, we found no patient without bacterial colonization, so antibiotic treatment based on urine cultures and resistance profiles is mandatory. SG placement in an infectious site is still critical. Nevertheless, UAF typically requires urgent treatment. Although there is no established consensus, it is recommended that these patients receive perioperative antibiotic treatment (Hong et al., 2016). Titomihelakis et al. suggest antibiotics for 6 weeks after the procedure, and in most cases lifelong antibiotics may not be unreasonable. Taking the patient’s medical condition into account, removal of the infected foreign body might be suitable (Titomihelakis et al., 2019).

**Fig. 2:**
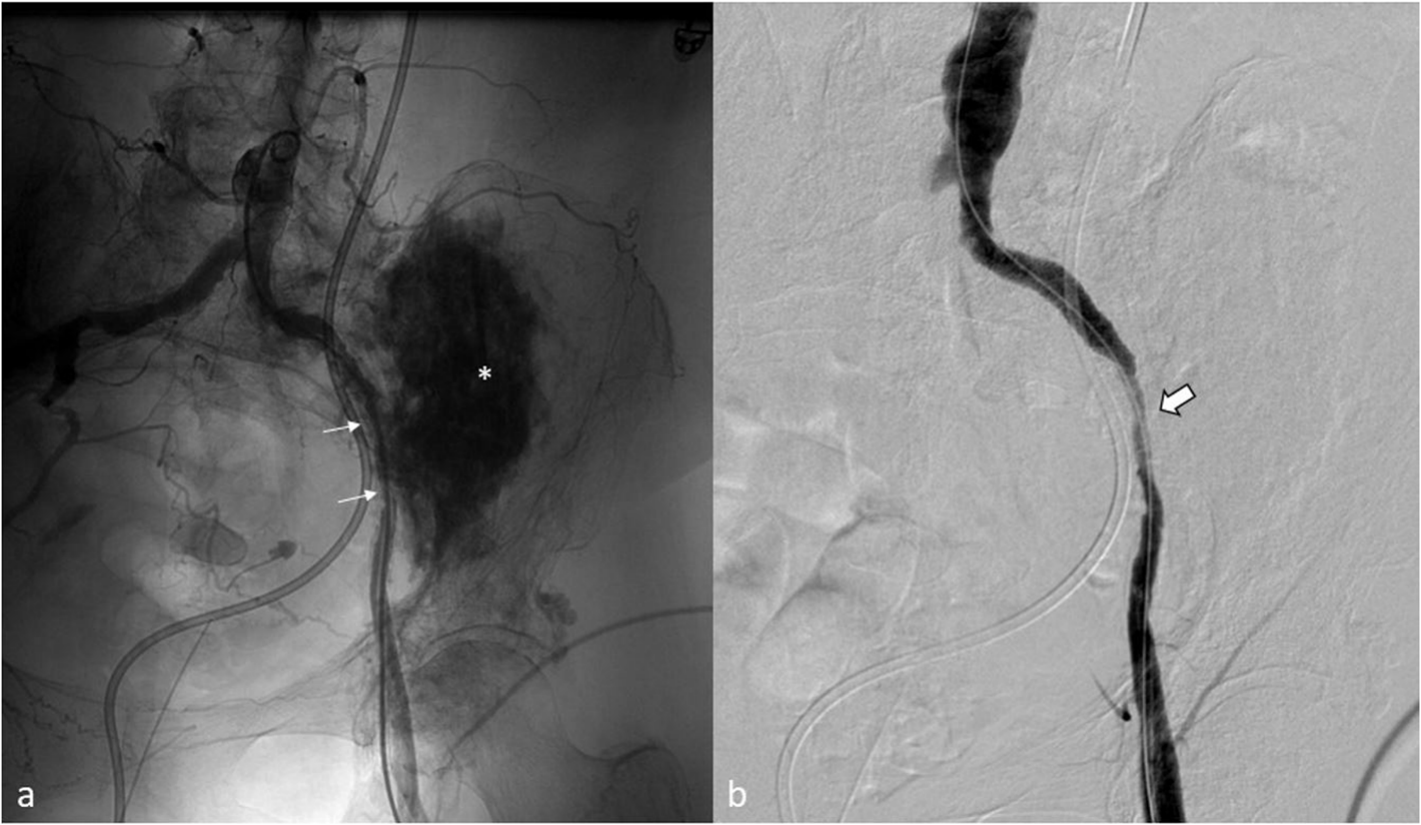
Case #3: 81-year-old woman with left sided bleeding from UAF. Angiogram shows the ureteric stent in direct proximity of the left CIA and EIA with closed IIA while there is no direct proof of the fistula on angiography. Massive calcified bone metastasis (*) compresses the left iliac arteries (small arrows) (**a**). The final control angiogram shows the crossing of the common iliac artery and the ureteric stent covered by a stent graft. Metastatic compression led to a not fully unfolded stent graft (large arrow) (**b**)

In the current series, recurrent bleedings developed after insufficient coverage of the fistula during the initial intervention and were manageable by a secondary treatment. None of these patients received secondary surgical treatment for recurrent hemorrhage. Additional risk factors included complex underlying situations with severe vasculopathy or stenosing tumor burden. Following our experience, we suggest to cover the ureteral crossing from the CIA to the EIA by a SG with prior coil embolization of the IIA. IIA coil embolization prevents retrograde perfusion of the UAF through the gluteal arteries, but also lowers the risk of recurrent bleedings – eg., in cases of fistulas from the proximal IIA. Embolization of the IIA alone would not be sufficient for UAF treatment (Massmann et al., 2020). It is crucial to detect fistula recurrence. In our series recurrent hematuria happened in 4/17 cases (23.5%), one of them died due to recurrence of the UAF (5.9%). Three recurrent fistulas could successfully be treated in a revisional endovascular procedure. Our literature review found a fistula recurrence rate of 11.8% and five UAF related deaths (3.3%). Guntau et al. identified recurrent hematuria in one of eight patients (12.5%). This patient could also successfully be treated with a secondary endovascular treatment, a combination of mild dilatation of the native iliac artery and a short distal landing zone seemed to be the cause (Guntau et al., 2017). Van den Bergh et al. found in their review of various procedures for the treatment of UAF a recurrent fistula related mortality of 18 out of 139 patients (13%) (van den Bergh et al., 2009). In our cohort one patient died due to recurrent hemorrhage 229 days after initial treatment. This patient had a large pelvic tumor mass which led to external compression and consecutive stenosis of the iliac vessels. The inserted SG unfolded incompletely during the initial procedure and SG thrombosis occurred during the postinterventional course. This led to a secondary procedure to treat acute lower limb ischemia, which resulted in reopening of the UAF with consequent bleeding (Fig. 2). Even if open surgery can become necessary in some UAF patients, endovascular approach should be evaluated as first approach. Studies have shown the long term effectiveness of endovascular treatment. Although, reasons for fistula recurrence have been poorly reported (Bilbao et al., 2005; Subiela et al., 2018; van den Bergh et al., 2009). Noh et al. found in their patients infection, pseudaneurysm formation at the SG edge, and persistent irritation by a pre-existing artificial intervertebral disc predisposing factors for recurrence (Noh et al., 2020). Type 1 endoleak by not fully covering stent edges also seems a risk factor (Perrenoud et al., 2020).

Our study is limited by its retrospective nature and by the small number of cases. In addition, follow-up periods varied substantially.

## Conclusions

In view of the low incidence of this disease and continuously evolving diagnostic and therapeutic tools, it seems difficult to establish evidence-based recommendations for these patients. Nevertheless, both urologists and interventional radiologists seem to be well aware of the special features of this rare disease. Endovascular therapy offers high technical success and rapid bleeding control rates. Severe complications like SG occlusions or SG infections are rare but maintain a problem. Since nearly all patients carry ureteral stents, antibiotic treatment based on urine cultures and resistance profiles is mandatory. Based on the patient’s medical condition explantation of an infected SG can be considered. Too maintain the risk for SG occlusions as low as possible patients should be put on single anti platelet therapy. If SG thrombosis has occured, both interventional and surgical thrombectomy are well suited options.

In conclusion, endovascular therapy for UAF is a safe and effective treatment option to prevent acute hemorrhage-related death. Since UAF recurrence often results in fatal hemorrhage close and long follow-up is crucial to timely perform additional treatments if necessary.

## Data Availability

All data generated or analysed during this study are included in this published article.

## Abbreviations

CECT: Contrast-enhanced computed tomography
CIA: Common iliac artery
EIA: External iliac artery
Fr: French
IIA: Internal iliac artery
SG: Stent graft
UAF: Ureteroarterial fistula
UECT: Unenhanced computed tomography
